# A Novel Technique of Uterine Manipulation in Laparoscopic Pelvic Oncosurgical Procedures: “The Uterine Hitch Technique”

**DOI:** 10.1155/2010/836027

**Published:** 2010-02-14

**Authors:** S. P. Puntambekar, A. M. Patil, N. V. Rayate, S. S. Puntambekar, R. M. Sathe, M. A. Kulkarni

**Affiliations:** Department of Minimally Invasive Oncology, Galaxy Laparoscopy Institute, Above Ayurved Rasashala, 25-A Karve Road, Pune, Maharashtra 411030, India

## Abstract

*Aim*. To describe a new technique of uterine manipulation in laparoscopic management of pelvic cancers. *Material and Methods*. We used a novel uterine hitch technique in 23 patients from May 2008 to October 2008. These patients underwent pelvic oncologic surgery including laparoscopic radical hysterectomy (*n* = 7), laparoscopic anterior resection (*n* = 4), laparoscopic abdominoperineal resection (*n* = 3), laparoscopic posterior exenteration (*n* = 4), or laparoscopic anterior exenteration (*n* = 5). The uterus was hitched to the anterior abdominal.wall by either a single suture in the fundus or by sutures through the round ligaments. *Results*. The uterine hitch technique was successfully accomplished in all procedures. It was performed in less than 5 minutes in all cases. It obviated the need for vaginal manipulation. An extra port for retraction could be avoided. There were no intraoperative complications. *Conclusion*. A practical, cheap and reproducible method for uterine manipulation, during pelvic oncologic surgery is described. It improves the stability of the uterus and also obviates the need for keeping an additional assistant for vaginal manipulation in any of the procedures.

## 1. Introduction

Uterine manipulation is essential for laparoscopic procedures involving dissection of the female pelvis. Several uterine manipulators are available to help manoever the uterus facilitation proper dissection in the pelvis. 

At our institute, we routinely use the myoma screw for uterine manipulation during total laparoscopic hysterectomy or surgery for cervical [1], bladder, and rectal cancers. 

However its use is avoided in two situations. First, in endometrial cancer, where peritoneal spread of tumor cells is feared. Second, in patients with pyometra in cervical cancer, where insertion of the screw would lead to spillage of pus into the abdominal cavity. 

Various vaginal manipulators are used in laparoscopic surgery to manipulate the uterus. When these enter through the cancer tissue, like the cervical canal or the endometrial cavity, manipulation can sometimes result in tissue breakup and bleeding. This can theoretically lead to cancer cells entering into the circulation and systemic spread. Some vaginal manipulators are comparatively expensive and require an additional assistant at the vaginal end. 

The uterus is often manipulated using a grasping forceps, but this entails use of one port for uterine manipulation or insertion of an additional port. Moreover, graspers often slip leading to tissue break up. This is especially common at the uterine cornua, which bleed during repeated manipulation with graspers. 

Hence, instead of a vaginal manipulator, a myoma screw, or a grasping forceps, we used a stitch to hitch up the uterus during laparoscopic pelvic oncosurgery. The uterus was hitched to the anterior abdominal wall by either a single suture in the fundus or by sutures through the round ligaments. This technique was very useful in patients requiring dissection in the puouch of Douglas as in cervical and rectal cancers.

When operating on endometrial cancer, the uterus was hitched from the round ligaments, thus facilitating its easy manipulation without spillage of cancer cells into the peritoneal cavity. 

Here we describe the uterine hitch technique for uterine manipulation, in patients who require radical pelvic dissection or in whom the use of uterine manipulators is contraindicated. We also describe the implications, advantages, and disadvantages of this technique.

## 2. Material and Methods

We performed the uterine hitch technique in 23 female patients who underwent laparoscopic pelvic oncosurgeries at Galaxy Laparoscopy Institute from May 2008 to October 2008. The procedures included laparoscopic radical hysterectomy for cervical cancer (*n* = 3) and for endometrial cancer (*n* = 4), laparoscopic anterior resection for rectal cancer (*n* = 2) and sigmoid cancer (*n* = 2), laparoscopic abdominoperineal resection for rectal cancer (*n* = 3) and laparoscopic posterior exenteration for cervical cancer invading rectum (*n* = 3) and rectal cancer invading uterus (*n* = 1) and laparoscopic anterior exenteration for bladder cancer (*n* = 2) and cervical cancer invading bladder (*n* = 3) (Table 1).

The uterine hitch technique was used in all the patients who required a radical pelvic dissection or in whom the use of a uterine manipulator was contraindicated (endometrial cancer and pyometra). 

The average age of the patients was 50 +/− 5 years. Previous surgeries included open tubal ligation (*n* = 20), caesarean section (*n* = 14), and open appendicectomy (*n* = 5). 

All patients underwent a standard preoperative preparation. Regional anesthesia, either spinal or epidural, was given in combination with general anaesthesia. A Foley catheter was placed into the bladder. The patient was placed in a modified Lloyd Davis position at approximately a 30 to a 45 degrees angle. 

A total of 4 or 5 ports were used (10 mm camera port through the umbilicus, a 10 mm port at the McBurney's point on right side, a 5 mm port pararectally at midclavicular line at level of umbilicus on the right side, and two 5 mm ports on left side, which were the mirror image of the right-sided ports). The left pararectal ports were used for insertion of the myoma screw. This port couls now be omitted when using the uterine hitch.

### 2.1. The Uterine Hitch Technique

**Figure 1:**
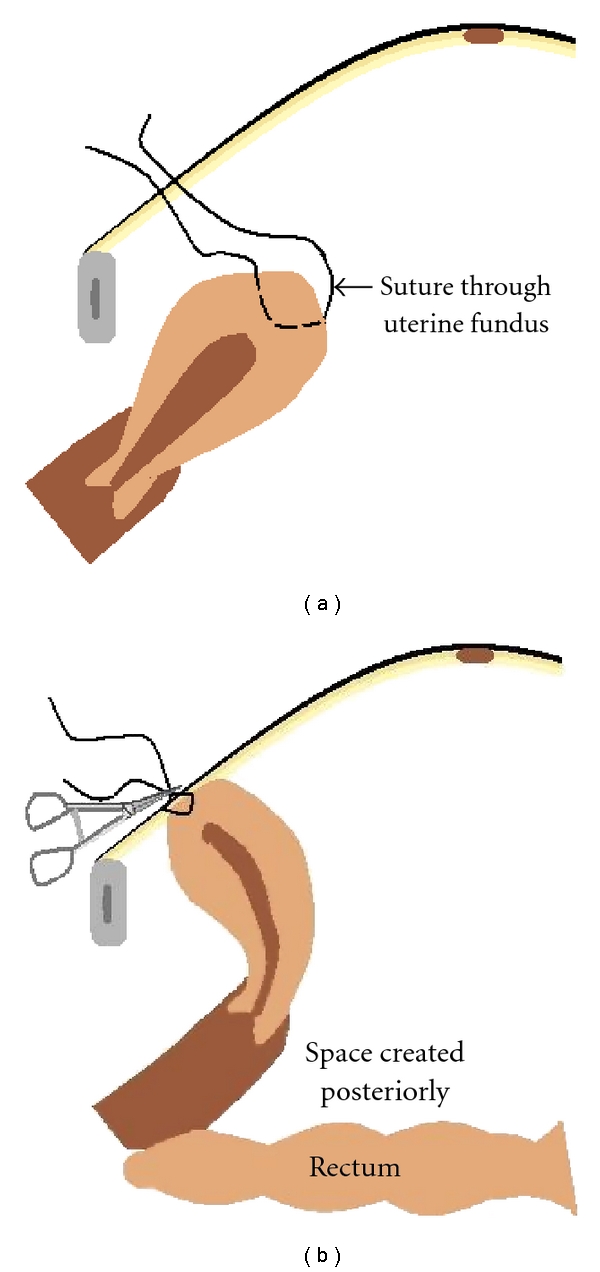
A diagrammatic representation of the uterine hitch technique. (a) Suture passed though the musculature of the uterine fundus. (b) The uterus is hitched to the anterior abdominal wall. A haemostatic forceps is applied to both at the ends. A pull on the sutures will antevert the uterus and displace it anteriorly.

Figure 1 illustrates the uterine hitch technique. 

**Figure 2:**
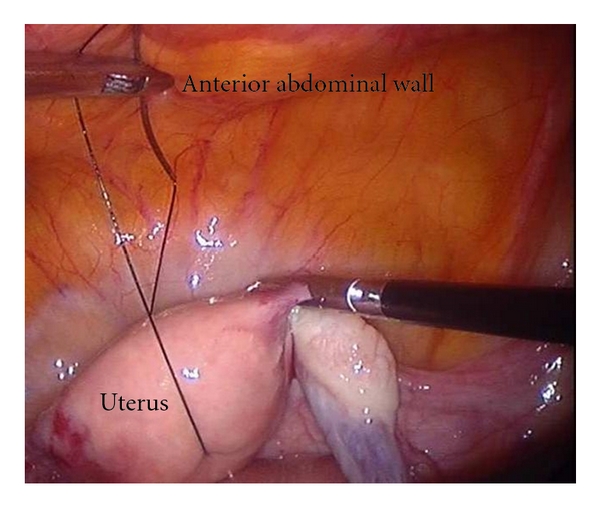
Monofilament polyamide suture on needle is used for hitching the uterus. The suture is passed through the uterine fundus anteroposteriorly and brought out to the exterior through the anterior abdominal wall.

The uterus was held using a nontraumatic grasper and brought towards anterior abdominal wall. Monofilament polyamide suture material, number 2, 0 on a straight needle (or on a curved needle that was partially straightened out), was inserted through the layers of the anterior abdominal wall at the level of the uterine fundus. This needle was held intraperitoneally with a laparoscopic needle holder and was passed anteroposteriorly, through the fundus of the uterus. The needle was then brought out of the abdomen just near the initial entry point (Figure 2). 

**Figure 3:**
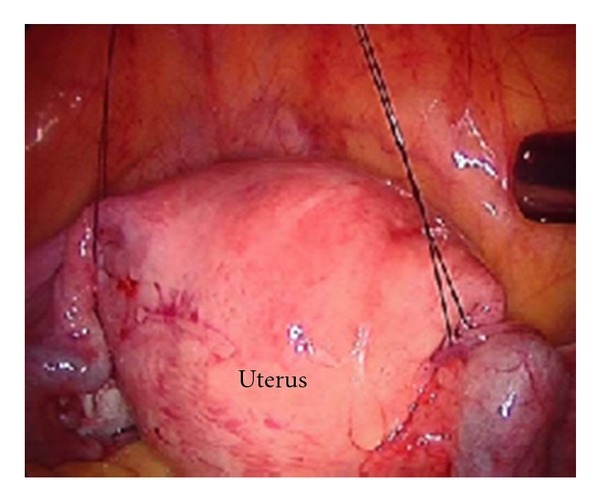
The uterus is suspended by two separate sutures through each of the round ligaments. This can be done in cases where the penetration of the uterus is against oncological principles, for example, in endometrial cancer.

Both threads were stretched and held with a haemostatic forceps close to the skin. In patients with endometrial cancer, the needle was not passed through the endometrial cavity. Instead, two separate sutures passing through each of the round ligament were used to suspend the uterus (Figure 3). 

**Figure 4:**
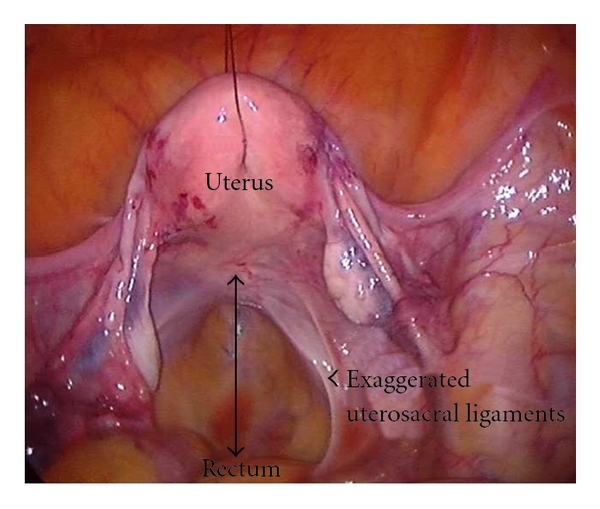
Hitching up the uterus improves the stability of the uterus. The uterosacral ligaments are stretched and this eases the posterior dissection.

Traction on the threads resulted in uterine anteversion and anterior displacement of the uterus. This stretched the uterosacral ligaments and provided additional space posteriorly for dissection in the pouch of Douglas and the pararectal spaces (Figure 4). 

During anterior dissection, the ends of the sutures were released, brought intraperitoneally, and then pulled cranially with a grasper. Cranial traction on the sutures facilitated dissection in the uterovesical or the prevesical space (Figure 4). Pulling on the sutures to the left or to right facilitated further lateral dissection.

## 3. Results

We have used the laparoscopic uterine hitch technique in 23 patients. The procedure was easily accomplished in all patients. The average time required for hitching up the uterus was less than 5 minutes. 

In one of the patients, the round ligament was torn due to the suture cutting through the tissue. In one of the obese patients, it was difficult to retrieve the needle through the abdomen, but it could be done by applying pressure on the abdomen when the needle was being removed. No other complication was noted. 

We did not need to use an extra uterine manipulator in any of the cases.

## 4. Discussion

Various uterine manipulators are available for use in laparoscopic pelvic surgeries. Though each one has its own advantages and disadvantages, none of the manipulators has all the attributes of an ideal manipulator and can be universally used. 

Vaginal manipulators are being used successfully by some for laparoscopic pelvic oncosurgery. Ramirez et al. described the use of a modified vaginal manipulator in laparoscopic radical hysterectomy for cervical and endometrial cancer [2]. Similarly, Spirtos et al. used the uterine sound steri stripped to the tenaculum as a uterine manipulator for laparoscopic radical hysterectomy [3]. The vaginal manipulators require an extra staff member to maintain the instrument in the correct position. Adjustments in the retraction are not in direct control of the operating surgeon, who has to instruct the assistant at the vaginal end as to what type of retraction is required. In patients with cervical stenoses, use of a uterine sound and cervical dilatation increases the risk of perforation [4]. In patients of cervical carcinoma, there is added risk of tearing of the cervix and bleeding or migration of the tissue into the endometrial cavity and thus into the peritoneal cavity. 

Lim et al. have described that the use of a uterine manipulator with an intrauterine balloon during laparoscopic surgery for endometrial cancer might be associated with positive cytological conversion [5]. Possible explanations were retrograde seeding of the tumor cells into the peritoneal cavity and the spillage of the preexisted tumor cells between the isthmus and the fimbriae. 

We have an extensive experience of Laparoscopic radical hysterectomy, Laparoscopic anterior exenteration, and Laparoscopic total pelvic exenteration. We also have a high number of patients who undergo advanced laparoscopic pelvic colorectal and urological oncosurgeries. 

In our published series of laparoscopic radical hysterectomy for cervical cancer, we used the myoma screw for uterine manipulation [1]. But a myoma screw could not be used in cases of endometrial cancer or in uterus with pyometra, for risk of spillage of tumor cells or pus into the peritoneal cavity. 

To obviate the disadvantages of a uterine manipulator, a myoma screw, or a grasping forceps, we adapted a technique which is simple and easy. We have used laparoscopic uterine hitch technique for more than 6 months in 23 such patients. 

The main advantage of this technique was that the stitch used for manipulation does not go through the tumor tissue. Moreover the operating surgeon could directly control and adjust the retraction to his/her satisfaction (unlike the vaginal manipulation, in which the operating surgeon had to instruct the assistant off and on). This reduced the operative time and stress. 

Ample space for dissection in the posterior compartment of the pelvis was created when the uterus was hitched to the anterior abdominal wall. 

This hitch resulted in adequate anteversion and anterior displacement. Cranial traction on the sutures facilitated dissection anterior to the uterus. In this position, there was also enough scope for lateral countertraction and dissection on the lateral sides. 

The left-upper pararectal port that was originally utilized for the myoma screw could now be used for assistance in retraction of the rectum and in suctioning. In some patients we could even avoid the above port. 

The problems anticipated were tearing of the uterus, inadvertent bleeding from the uterus, breaking of the suture, accidental perforation of the bladder, or abdominal wall vessel. But apart from some difficulty in passing the suture through the abdominal wall in an obese patient and tearing of the round ligament in one patient, we did not face any other problem due to the uterine hitch. 

Similar techniques have been used previously for laparoscopy in benign conditions. Tsin and Colombero described the laparoscopic leash technique to prevent specimen loss during laparoscopy [6]. Giesler further extended this concept with a puppet string variation to facilitate traction and manipulation in large broad ligament fibroids [7]. The uterine hitch technique is an excellent alternative to routine manipulation techniques when operating laparoscopically on cancers in the pelvis.

## 5. Conclusion

The hitching of the uterus is an innovative method for manipulating the uterus in several laparoscopic pelvic oncosurgical procedures. It is a simple, effective, easy, and economical technique which facilitates dissection posterior to the uterus and in cases where routine uterine manipulators are relatively contraindicated (endometrial carcinoma, pyometra). It can also be used in advanced oncosurgical procedures to reduce the number of ports used by one or make available one port (which is, otherwise, used for uterine manipulation, using some kind of instrumentation), for dissection or retraction of organs other than the uterus. It improves the stability of the uterus and also obviates the need for keeping an additional assistant for vaginal manipulation in any of the procedures. 

##  Article Precis

An easy and innovative method of uterine manipulation in laparoscopic pelvic oncosurgery.

## Figures and Tables

**Table 1:** List of surgeries where the uterine hitch technique was used.

No.	Surgery	Indication	No. of cases
1	Laparoscopic Radical hysterectomy	Cervical cancer	3
		Endometrial cancer	4
2	Laparoscopic anterior resection	Rectal cancer	2
		Sigmoid cancer	2
3	Laparoscopic abdominoperineal resection	Rectal cancer	3
4	Laparoscopic posterior exenteration	Cervical cancer	3
		Rectal cancer	1
5	Laparoscopic anterior exenteration	Bladder cancer	2
		Cervical cancer	3
